# Synergism in drug combination of new tuberculosis antibiotics for drug-resistant *Mycobacterium tuberculosis in vitro*

**DOI:** 10.1128/spectrum.02128-25

**Published:** 2026-07-10

**Authors:** Run Huang, Yifan He, Jinghui Yang, Yidian Liu, Qin Sun, Ruoyan Ying, Wei Sha

**Affiliations:** 1 Department of Tuberculosis, Shanghai Pulmonary Hospital, School of Medicine, Tongji University 12476, Shanghai, People’s Republic of China; 2 Department of Clinical Laboratory, Shanghai Pulmonary Hospital, School of Medicine, Tongji University 12476, Shanghai, People’s Republic of China; 3 Shanghai Key Laboratory of Tuberculosis, Shanghai Pulmonary Hospital, School of Medicine, Tongji University 12476, Shanghai, People’s Republic of China

**Keywords:** tuberculosis, DR-TB, combined drug sensitivity, FICI, MIC

## Abstract

The treatment of drug-resistant tuberculosis (DR-TB) faces significant challenges. Novel antituberculosis drugs such as bedaquiline (BDQ), pretomanid (Pa824), and delamanid (DLM) offer a promising treatment for DR-TB. However, the synergistic effects of these drugs have been insufficiently explored. In this research, we evaluated the synergistic effects of two dual-drug combinations (BDQ-Pa824 and BDQ-DLM) and four triple-drug combinations (BDQ-DLM-clofazimine [CFZ], BDQ-DLM-linezolid [LZD], BDQ-Pa824-CFZ, and BDQ-Pa824-LZD) against 30 *Mycobacterium tuberculosis* (*MTB*) strains isolated from TB patients. The results indicated that the synergistic antimycobacterial effects of BDQ-DLM-LZD and BDQ-Pa824-LZD show no statistically significant difference, suggesting that BDQ-DLM-LZD could be considered a potential therapeutic alternative for DR-TB. This provides evidence for the development of more flexible treatment options for DR-TB.

**IMPORTANCE:**

The rapid emergence of antimicrobial resistance in *Mycobacterium tuberculosis* (MTB) presents a critical challenge for global public health. Given that the synergistic effects of antituberculosis drugs against drug-resistant tuberculosis have not been systematically analyzed. In this research, we evaluated the synergistic antimycobacterial efficacy of the new optimized antituberculosis regimen (BDQ-DLM-LZD or BDQ-DLM-CFZ) and the standard regimen (BDQ-Pa824-LZD or BDQ-Pa824-CFZ) for TB strains. This highlights the therapeutic substitutability of BDQ-DLM or BDQ-Pa824-based triple-drug regimens for TB strains.

## INTRODUCTION

Tuberculosis (TB) has once again emerged as the leading cause of death due to an infectious disease (1). Both multidrug-resistant tuberculosis (MDR-TB) and rifampicin-resistant tuberculosis (RR-TB) continue to pose a significant public health crisis. As of 2024, treatment success rates for MDR/RR-TB have improved; however, of the estimated 390,000 individuals who developed MDR/RR-TB, only 42% received proper diagnosis and effective treatment (2). Compared to those with drug-sensitive TB, patients with MDR/RR-TB experience a longer treatment course, higher treatment costs, and poorer treatment outcomes (3).

Major improvements have been achieved in the treatment of drug-resistant tuberculosis in recent years, with two new drug classes—diarylquinolines (bedaquiline) and nitroimidazoles (pretomanid and delamanid)—being central to this success (4, 5). Bedaquiline (BDQ), a diarylquinoline-class antitubercular agent, exerts its bactericidal effect by selectively inhibiting the ATP synthase of *MTB*.^6^ Both delamanid (DLM) and pretomanid (Pa824) are prodrugs that require reductive activation by the deazaflavin (F420)-dependent nitroreductase (Ddn) in *MTB*. Their activated forms inhibit the biosynthesis of mycolic acids, which are essential components of the bacterial cell wall (7). Linezolid (LZD) is a protein synthesis inhibitor that works by binding and blocking the peptidyl transferase center on the 50S ribosomal subunit and disrupts bacterial protein synthesis (8). Clofazimine (CFZ) is an antituberculosis drug with multiple mechanisms of action, such as inhibiting the synthesis of mycolic acid and protein in mycobacteria (5). It plays an important role in the treatment of MDR/RR-TB, especially when combined with BDQ and DLM, enhancing treatment efficacy (9).

In 2024, the World Health Organization (WHO) recommended the use of a new 6-month treatment regimen composed of BDQ, DLM, LZD (600 mg), levofloxacin (Lfx), and CFZ (6BDLLfxC), as well as three 9-month all-oral regimens (specifically BLMZ, BLLfxCZ, and BDLFxZ) for the treatment of MDR/RR-TB patients who are resistant to or sensitive to fluoroquinolones (FQs) (10). With the rapid increase in resistance and cross-resistance rates to BDQ (11) and consecutively to other drugs in the regimen (12), the risk of ineffective treatment and transmission of resistant strains cannot be understated.

Although previous research has reported the *in vitro* activity and synergy among different antimicrobial agents against *MTB* strains (13), the synergistic effects of BDQ, DLM, Pa824, CFZ, and LZD against MDR/RR-TB strains have not been systematically analyzed. To more accurately assess the effectiveness of the new treatment regimen, 30 *MTB* strains from our preserved strain library were selected for this study. We evaluated the efficacy of the new optimized antituberculosis regimen (BDQ-DLM-LZD or BDQ-DLM-CFZ) for MDR/RR-TB strains by calculating the fractional inhibitory concentration index (FICI) for each bacterial strain. Furthermore, we compared the *in vitro* bacterial inhibition effect of the new regimen (BDQ-DLM-LZD or BDQ-DLM-CFZ) with that of the standard regimen (BDQ-Pa824-LZD or BDQ-Pa824-CFZ). Finally, we assessed the regimens' ability to inhibit the growth of organisms using a macrophage model.

## MATERIALS AND METHODS

### *M. tuberculosis* isolates

This study included 30 clinical isolates obtained from our preserved strain library, including 20 MDR-TB, 5 RR-TB, and 5 drug-sensitive (DS-TB) *MTB* isolates, at Shanghai Pulmonary Hospital affiliated to Tongji University in Shanghai, China, between January 2023 and December 2024. These 30 clinical isolates reflect the local epidemiological profile of DR-TB in Shanghai, China. Drug susceptibility results of the 30 isolates were obtained by the proportion method using the BACTEC MGIT 960 system. The *MTB* H37Rv (number: ATCC27294) was used as a reference strain. These isolates were preserved in 7H9 broth (Becton Dickinson, Franklin Lakes, NJ) with 15% glycerol in a freezer at −80°C until analysis. To prepare for drug susceptibility testing, the test isolates were cultured at 37°C in Middlebrook 7H9 broth (Becton Dickinson) supplemented with 10% ADC (5% bovine serum albumin [BSA], 2% dextrose, 5% catalase), and 0.05% Tween-80 (Sigma-Aldrich) until they reached mid-log phase optical density (OD) (OD_590_ ≈ 0.4, ~2.5 × 10^8^ CFU/mL). The culture was then diluted to approximately ~10^5^ CFU/mL for testing.

### Antimicrobial agents

BDQ, DLM, CFZ, and LZD were purchased from Med Chem Express. Pa824 was obtained from Beyotime. Drug configuration: All drugs were weighed to 10 mg; among BDQ, DLM, Pa824, CFZ, and LZD, dry powder was prepared with 1 mL of dimethyl sulfoxide (DMSO) to a concentration of 10 mg/mL, respectively; all drug solutions were filtered and sterilized with a 0.22 µm sterile filter, and stored separately in a freezer at −80°C. Further dilutions were made with a coating buffer containing 30% ethanol and 0.5% sucrose.

### Drug susceptibility testing

Broth microdilution MIC testing was conducted for five anti-TB agents, including BDQ, DLM, Pa824, CFZ, and LZD. This was performed according to previously described previously (14, 15). Serial double dilutions of each antimicrobial agent were prepared for each isolate, using the following ranges of drug concentration: 0.03–2 µg/mL for BDQ, 0.0015–2 µg/mL for DLM, 0.0015–2 µg/mL for Pa824, 0.5–4 µg/mL for CFZ, and 2–16 µg/mL for LZD. This range covers the clinical thresholds established by the Clinical and Laboratory Standards Institute (CLSI) M24, 7th Edition guidelines for CFZ and LZD (CFZ, S ≤ 0.5 µg/mL; LZD, S ≤ 1.0 µg/mL) (16, 17), while also incorporating the epidemiological cutoff values for BDQ (ECV ≈ 0.25 µg/mL) and DLM (ECOFF ≈ 0.016 µg/mL) from CLSI M62 and EUCAST (18–20). For Pa824, the concentration range encompasses the widely reported wild-type MIC range and the consensus ECV (0.25–0.5 µg/mL) documented in the literature. Adjustments were made based on the growth. In brief, if the standard highest concentration of a drug failed to inhibit the growth of the tested isolates, the highest concentration was doubled. Conversely, if the tested isolates could not grow at the standard lowest concentration of a drug, the lowest concentration was halved. The MIC test was conducted in duplicate to ensure the reliability of the results.

The checkerboard titration method was used to test the combinations of BDQ with DLM, BDQ with Pa824, BDQ and DLM combined with CFZ or LZD, and BDQ and Pa824 combined with CFZ or LZD. For the combination of two agents, such as BDQ (drug A) with DLM (drug B), DLM was serially diluted along the abscissa, while BDQ was diluted along the ordinate. A1, H12 wells to a 96-well plate (rows A–H, columns 1–12) were prepared as a growth control with a drug-free medium. The combination of BDQ with Pa824 was prepared as BDQ with DLM. Three-drug combination checkerboard titration methods were principally based on the standard two-drug (BDQ and DLM or BDQ and Pa824) combination checkerboard assay. Two-drug combination checkerboard plates were prepared by dispensing the serially diluted DLM along the abscissa and Pa824 along the ordinate in a 96-well plate. The third drug (CFZ or LZD) was then dispensed on the whole plate with one of the serial concentrations from the MIC range, except the A1 growth control well, as shown in Table 1. To prepare the drug plates, sterile 96-well plates were added with a total volume of 20 µL of drug-containing coating buffer using an automatic dispenser. For the drug susceptibility testing (DST), 200 µL of mycobacterial suspension was added to each plate. Following an incubation period of 2 weeks at 37°C, we first observed the growth status of the drug control strain (H37Rv) and the growth control wells (wells A1 and H12 in the dual-drug plates, and well A1 in the triple-drug plates) to ensure that the drugs were effective and that each strain grew normally. After confirming this, we recorded the minimum inhibitory concentrations (MICs) of all agents in the various combinations.

**TABLE 1:** The checkerboard titration arrangement on 96-well plates for triple-drug susceptibility testing^*a*^

1	1	2	3	4	5	6	7	8	9	10	11	12
A	No drug	Dlm0.0015	Dlm0.003	Dlm0.0075	Dlm0.015	Dlm0.03	Dlm0.06	Dlm0.125	Dlm0.25	Dlm0.5	Dlm1	Dlm2
B	Bdq0.03	B0.03 + D.0015	B0.03 + D.003	B0.03 + D.0075	B0.03 + D.015	B0.03 + D.03	B0.03 + D.06	B0.03 + D.125	B0.03 + D.25	B0.03 + D.5	B0.03 + D1	B0.03 + D2
C	Bdq0.06	B0.06 + D.0015	B0.06 + D.003	B0.06 + D.0075	B0.06 + D.015	B0.06 + D.03	B0.06 + D.06	B0.06 + D.125	B0.06 + D.25	B0.06 + D.5	B0.06 + D1	B0.06 + D2
D	Bdq0.125	B0.125 + D.0015	B0.125 + D.003	B0.125 + D.0075	B0.125 + D.015	B0.125 + D.03	B0.125 + D.06	B0.125 + D.125	B0.125 + D.25	B0.125 + D.5	B0.125 + D1	B0.125 + D2
E	Bdq0.25	B0.25 + D.0015	B0.25 + D.003	B0.25 + D.0075	B0.25 + D.015	B0.25 + D.03	B0.25 + D.06	B0.25 + D.125	B0.25 + D.25	B0.25 + D.5	B0.25 + D1	B0.25 + D2
F	Bdq0.5	B0.5 + D.0015	B0.125 + D.003	B0.5 + D.0075	B0.5 + D.015	B0.5 + D.03	B0.5 + D.06	B0.5 + D.125	B0.5 + D.25	B0.5 + D.5	B0.5 + D1	B0.5 + D2
G	Bdq1	B1 + D.0015	B1 + D.003	B1 + D.0075	B1 + D.015	B1 + D.03	B1 + D.06	B1 + D.125	B1 + D.25	B1 + D.5	B1 + D1	B1 + D2
H	Bdq2	B2 + D.0015	B2 + D.003	B2 + D.0075	B2 + D.015	B2 + D.03	B2 + D.06	B2 + D.125	B2 + D.25	B2 + D.5	B2 + D1	CFZ/LZD

^*a*^ The final concentrations of CFZ added to the whole plate except well A1 were 0.5, 1, 2, and 4 μg/mL, while the corresponding final concentrations of LZD were 2, 4, 8, and 16 μg/mL, respectively.

The FICI of two drugs was calculated by the formula FICI = (MIC A + B/MIC A) + (MIC B + A/MIC B), where MIC A + B represents the MIC of compound A when combined with B; MIC B + A, the MIC of compound B when combined with A; and MIC A and MIC B, the MIC of compounds A and B tested alone, respectively. A FICI value ≤0.5 was considered to indicate synergism, a FICI value between 0.5 and 4 was considered indifferent, and a FICI value >4 was considered to reflect antagonism.

The FICI of three drugs was calculated by the formula FICI = (MIC A + B + C/MIC A) + (MIC B + A + C/MIC B) + (MIC C + A + B/MIC C), where MIC A + B + C represents the MIC of compound A when combined with B and C; MIC B + A + C, the MIC of compound B when combined with A and C; MIC C + A + B, the MIC of compound C when combined with A and B; and MIC A, MIC B, and MIC C, the MICs of compounds A, B, and C tested alone, respectively. A FICI value ≤0.75 was considered to indicate synergism, a FICI value between 0.75 and 4 was considered indifferent, and a FICI value >4 was considered to reflect antagonism.

### Colony-forming unit (CFU) assay

We established a macrophage model by differentiating a human monocytic cell line (THP-1) into macrophages using Phorbol 12-myristate 13-acetate (PMA). For this experiment, we plated 500 μL (1 × 10^5^ cells/well) of THP-1 cells in 24-well plates, suspended in RPMI 1640 medium supplemented with 10% fetal bovine serum (FBS) and PMA at a concentration of 100 ng/mL. After 48 h, THP-1 cells differentiated into macrophages that adhered to the bottom of the wells. Next, we prepared a bacteria solution with a turbidity of OD_590_ ≈ 0.4 (~2.5 × 10^8^ CFU/mL), which was then diluted to a final concentration of 5 × 10^5^ CFU/well, achieving a multiplicity of infection of 5. After 3 h of infection at 37℃ in a 5% CO_2_–95% air atmosphere, we removed the supernatant and washed the wells three times with phosphate-buffered saline (PBS) to eliminate any extracellular bacteria. Following this, we added 500 μL of well-fresh RPMI 1640 medium with 10% FBS or one of four different regimens, which included BDQ-DLM-LZD, BDQ-DLM-CFZ, BDQ-Pa824-LZD, or BDQ-Pa824-CFZ. The drug concentrations used were: 0.125 µg/mL for BDQ, 0.05 µg/mL for DLM, 0.05 µg/mL for Pa824, 0.5 µg/mL for CFZ, and 1 µg/mL for LZD.

After the treatment, the untreated control group (NC) was given medium and 10% FBS alone. Following a period of either 24 h or 7 days, the cells were washed three times with PBS. Then, 500 μL of 1% Triton X-100 was applied to each well for 10 min. The lysate was serially diluted to 10^−1^, 10^−2^, and 10^−3^, and 50 μL of each dilution was spread onto 7H10 agar containing ampicillin (10 µg/mL). The plates were incubated at 37℃ in a 5% CO_2_–95% air atmosphere for 4 weeks. After the incubation period, the number of CFU of MTB isolates on each plate was counted.

### Statistical analyses

All experimental data were analyzed using GraphPad Prism software. The MIC_50_ and MIC_90_ were used to describe the central tendency and dispersion of the data, and the statistical significance of differences between the groups was determined using the Wilcoxon-paired test. The synergistic effects of the dual-drug combination and the three-drug combination were quantified using the FICI value. Chi-squared test or Fisher’s exact test was used to analyze the distribution of FICI values. *P*-value < 0.05 was considered statistically significant.

## RESULTS

### Dual-drug susceptibility testing of the combinations of BDQ and DLM, BDQ, and Pa824

**Fig 1:**
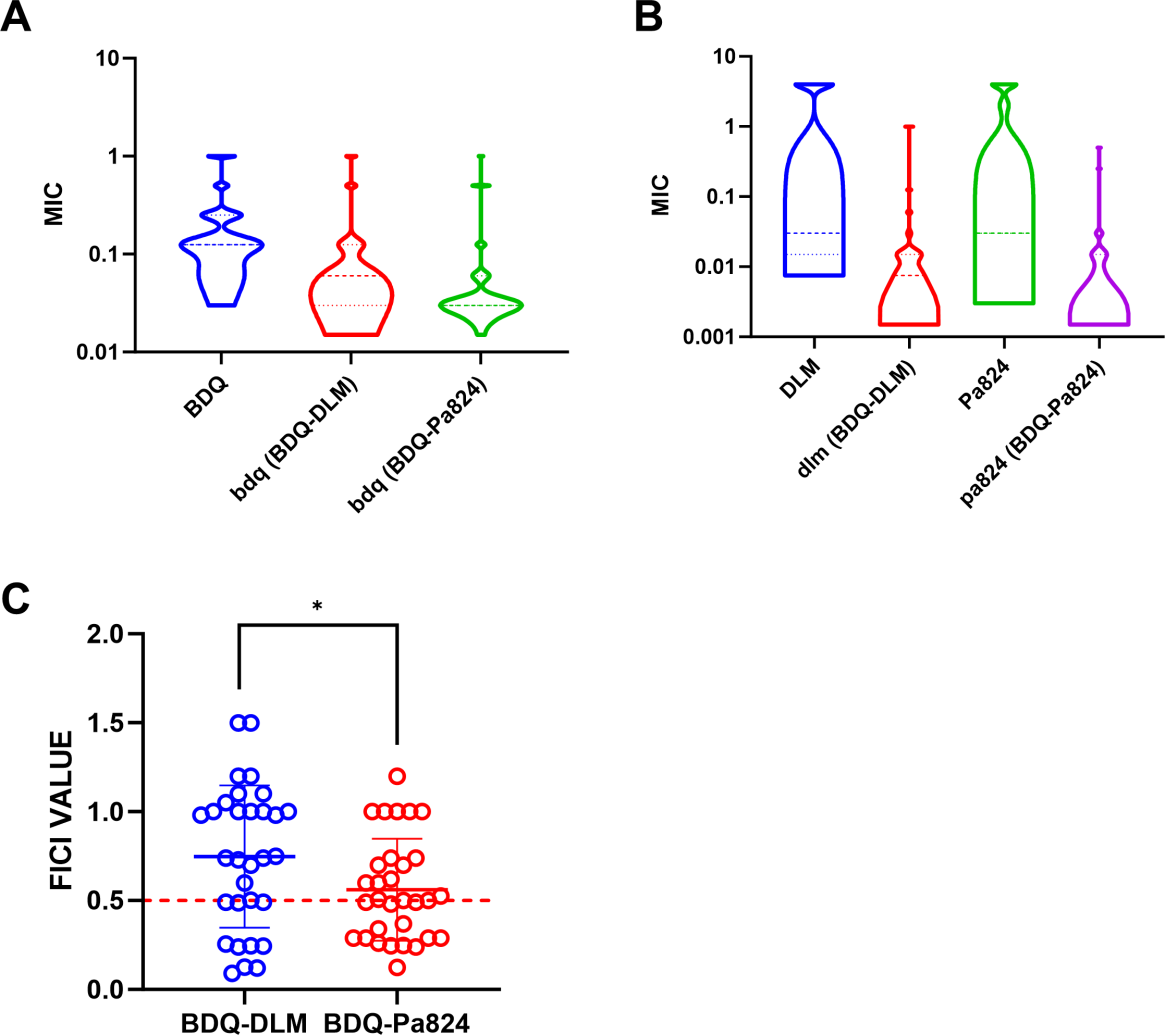
Comparison of MIC (**A and B**) and FICI (**C**) results from dual-drug susceptibility testing against MTB. MIC, minimal inhibitory concentration; FICI, the fractional inhibitory concentration index; BDQ, MIC of BDQ tested alone; bdq, MIC of BDQ tested with the combination of DLM or Pa824; DLM, MIC of DLM tested alone; dlm, MIC of DLM tested with the combination of BDQ; Pa824, MIC of Pa824 tested alone; pa824, MIC of Pa824 tested with the combination of BDQ; * indicates that the *P* values were statistically significant.

The single- and dual-drug susceptibility test results for the three antimicrobial agents and two combinations against 30 *MTB* clinical strains isolated from TB patients are shown in Table S1 and Fig. 1A and B. The MIC_50_ and MIC_90_ of BDQ, DLM, and Pa824 were all decreased after the combination with the other drug (*P* < 0.001), which suggests that the combinations of BDQ and DLM or Pa824 exerted synergistic effects with each other’s antimicrobial activity. After BDQ was combined with Pa824, the MIC_50_ and MIC_90_ of BDQ were reduced from 0.125 to 0.03 mg/L and 1 to 0.5 mg/L (*P* < 0.001), respectively. Moreover, the MIC_50_ and MIC_90_ of Pa824 were reduced from 0.03 to 0.0015 mg/L and 4 to 0.03 mg/L (*P* < 0.001), respectively. After BDQ was combined with DLM, the MIC_50_ and MIC_90_ of BDQ also decreased after it was combined with DLM, from 0.125 to 0.06 mg/L and 1 to 0.5 mg/L (*P* < 0.001), respectively. The MIC_50_ and MIC_90_ of DLM were reduced from 0.03 to 0.0075 mg/L and 4 to 0.06 mg/L (*P* < 0.001), respectively. The above results indicated that BDQ has a significant synergistic effect on DLM or Pa824.

The synergistic, indifferent, or antagonistic effects of each drug combination on the clinical isolates are shown in Table S2 and Fig. 1C. FICI analysis revealed that the FICI values of the combination of BDQ with Pa824 were significantly lower than those of the combination of BDQ with DLM (*P* = 0.0393 and *P* < 0.05, respectively). Furthermore, the combination of BDQ with Pa824 exhibited synergistic and indifferent rates of 43.3% and 56.7%, respectively, while the combination of BDQ with DLM showed rates of 36.7% and 63.3%, respectively. Notably, neither of the two dual-drug combinations demonstrated any antagonistic effects. These results suggested that the combination of BDQ with Pa824 was more likely to show synergy than that of BDQ with DLM. Due to the differing synergistic effects observed with two dual-drug combinations, we further investigated the effects of three-drug combinations based on the combinations of BDQ with DLM or BDQ with Pa824.

### Triple-drug susceptibility testing (based on the combination of BDQ with DLM or BDQ with Pa824)

**Fig 2:**
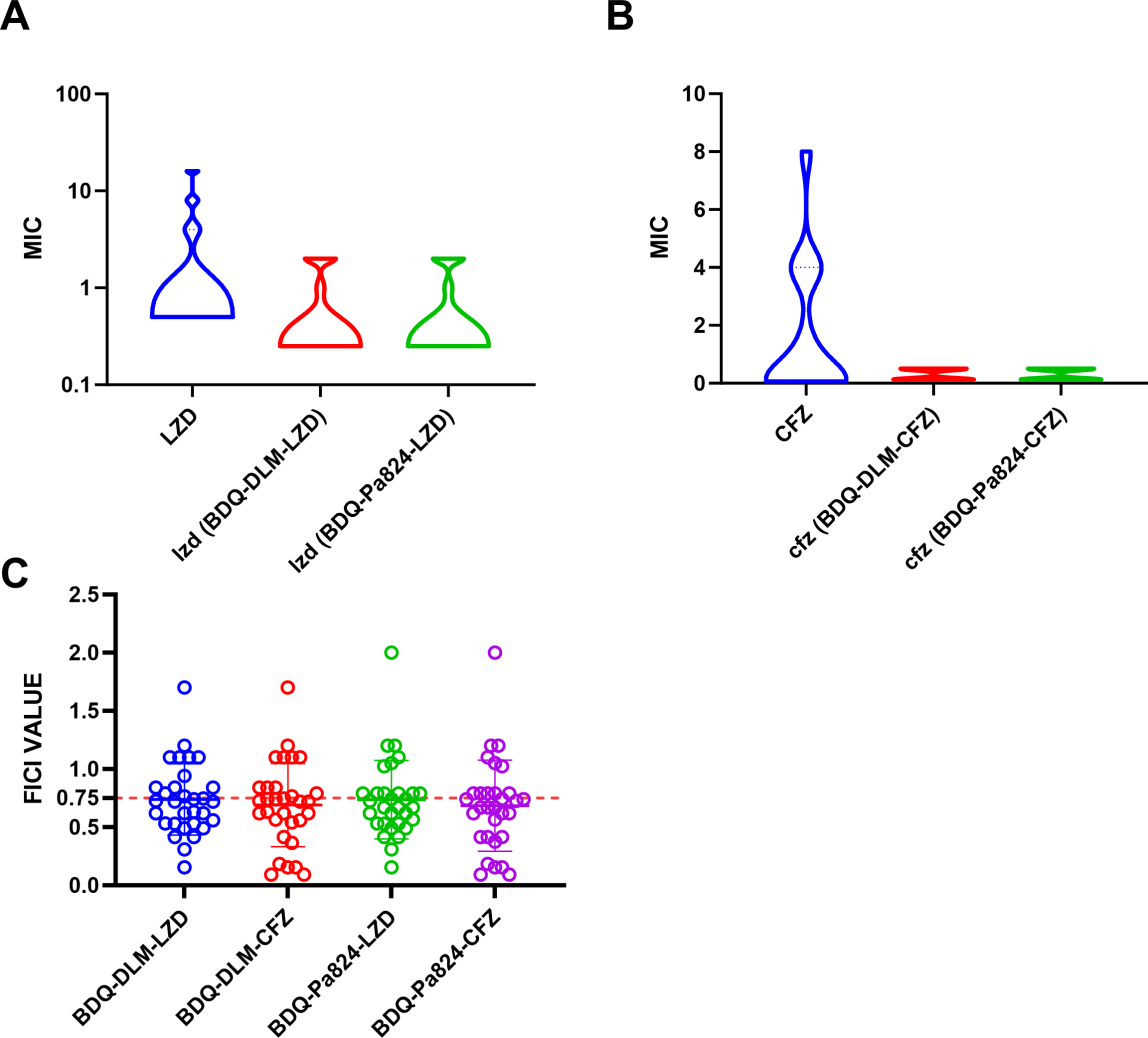
Comparison of MIC (**A and B**) and FICI (**C**) results from triple-drug susceptibility testing against MTB. MIC, minimal inhibitory concentration; FICI, the fractional inhibitory concentration index; LZD, MIC of LZD tested alone; lzd, MIC of LZD tested with the combination of BDQ and Pa824 or BDQ and DLM; CFZ, MIC of CFZ tested alone; cfz, MIC of CFZ tested with the combination of BDQ and Pa824 or BDQ and DLM.

The dual-drug susceptibility testing provided stronger statistical evidence for synergistic effects for the combinations of BDQ with DLM and BDQ with Pa824. We thus tested the individual MICs of CFZ and LZD in the three-drug combinations. As shown in Table S3 and Fig. 2A and B, the combination of LZD with BDQ and DLM decreased the MIC_90_ of LZD from 8 to 2 mg/L (*P* < 0.001), and the combination of LZD with BDQ and Pa824 decreased the MIC_90_ of LZD from 8 to 2 mg/L (*P* < 0.001). The combination of CFZ with BDQ and DLM decreased the MIC_90_ of CFZ from 4 to 0.5 mg/L (*P* < 0.001), and the combination of CFZ with BDQ and Pa824 decreased the MIC_90_ of LZD from 4 to 0.5 mg/L (*P* < 0.001).

Of the 30 *MTB* strains tested, the levels of synergism between four three-drug combinations, BDQ and DLM or BDQ and Pa824 combined with CFZ or LZD, were similar (BDQ-DLM-LZD vs. BDQ-DLM-CFZ, *P* = 0.9476; BDQ-DLM-LZD vs. BDQ-Pa824-LZD, *P* > 0.9999; BDQ-DLM-LZD vs. BDQ-Pa824-CFZ, *P* = 0.9292; BDQ-DLM-CFZ vs. BDQ-Pa824-LZD, *P* = 0.9531; BDQ-DLM-CFZ vs. BDQ-Pa824-CFZ, *P* > 0.9999; BDQ-Pa824-LZD vs. BDQ-Pa824-CFZ, *P* = 0.9358), as shown in Table S4 and Fig. 2C.

### CFU assays

**Fig 3:**
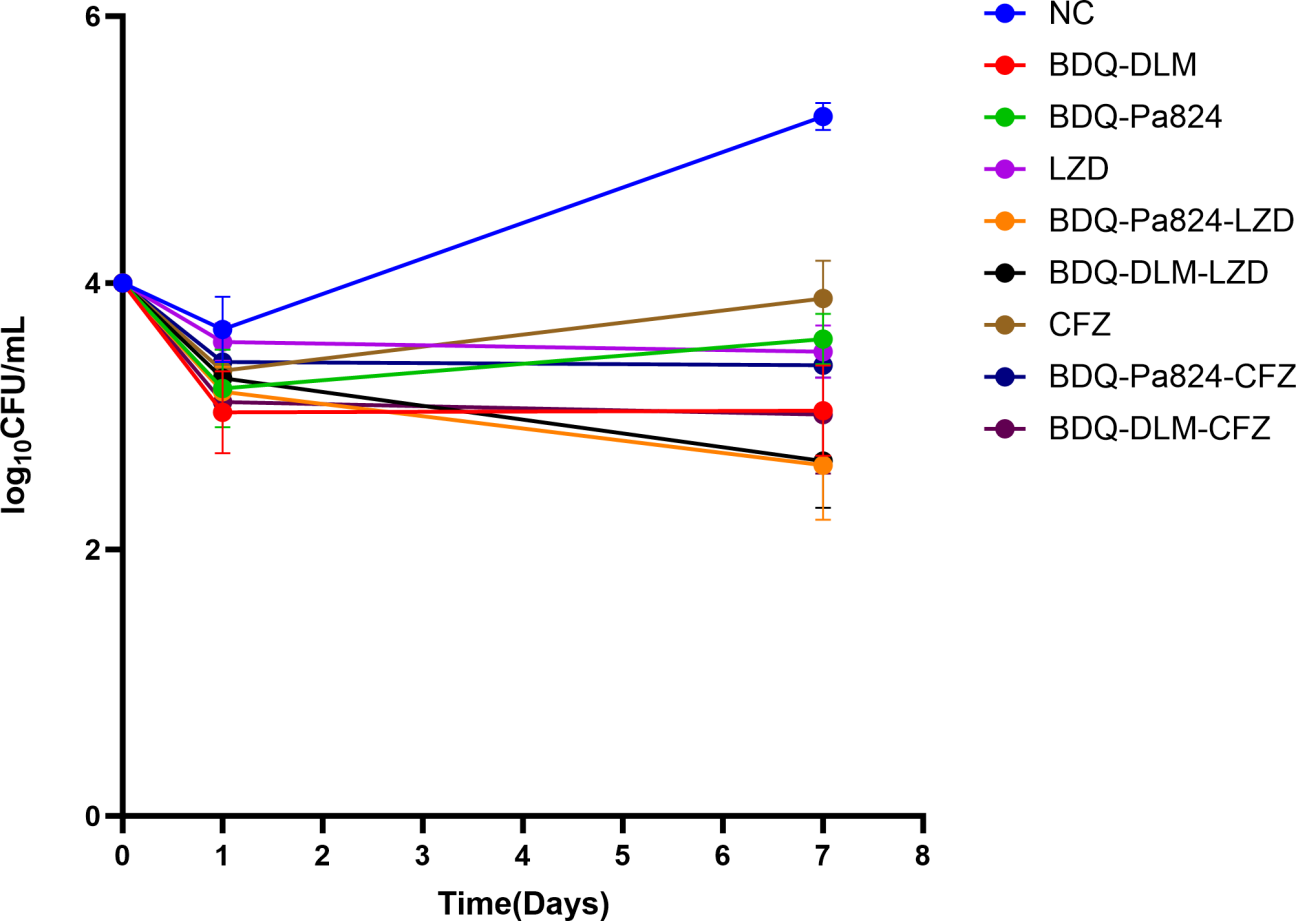
Comparison of CFU for each drug before and after combinations from dual-drug or triple-drug susceptibility testing. CFU, A colony-forming unit; NC, control group.

To quantify the impact of different drug combinations on the intracellular proliferation of *MTB*, we observed the CFU values in various treatment groups over a period of 1 day and 7 days, measured in units of log_10_ CFU/mL. As shown in Table S5 and Fig. 3, the NC group, as a negative control, maintained the baseline CFU, confirming the active growth of bacteria without drug intervention. All groups began with initial bacterial counts of 4 log_10_ CFU/mL on day 0. After 1 day of treatment, all experimental groups showed a significant reduction in CFU values. The CFU value of NC was reduced from 4 to 3.65 log_10_ CFU/mL. Additionally, the CFU values of LZD or CFZ decreased from 4 to 3.56 log_10_ CFU/mL and 4 to 3.34 log_10_ CFU/mL, respectively, and the CFU values of the combinations of BDQ with DLM or BDQ with Pa824 were reduced from 4 to 3.03 log_10_ CFU/mL and 4 to 3.21 log_10_ CFU/mL, respectively. Furthermore, the CFU values of the combinations of LZD and BDQ with Pa824 or DLM decreased from 4 to 3.18 log_10_ CFU/mL and 4 to 3.29 log_10_ CFU/mL, respectively, and the CFU values of the combinations of CFZ and BDQ with Pa824 or DLM were reduced from 4 to 3.41 log_10_ CFU/mL and 4 to 3.11 log_10_ CFU/mL, respectively.

Specifically, the CFU value of the NC group increased from 3.65 to 5.25 log_10_ CFU/mL after an additional 6 days of treatment. During this period, the CFU value of LZD decreased from 3.56 to 3.48 log_10_ CFU/mL, whereas that of CFZ increased from 3.34 to 3.88 log_10_ CFU/mL on day 7. Meanwhile, the CFU value of the combination of BDQ with DLM remained stable, ranging from 3.03 to 3.04 log_10_ CFU/mL and that of the combination of BDQ with Pa824 was increased from 3.21 to 3.58 log_10_ CFU/mL. In the three-drug combinations, the CFU value of the combination of LZD and BDQ with Pa824 was reduced from 3.18 to 2.63 log_10_ CFU/mL, and that of the combination of LZD and BDQ with DLM decreased from 3.29 to 2.66 log_10_ CFU/mL. Additionally, the CFU values of the combinations of CFZ and BDQ with Pa824 or DLM were reduced from 3.41 to 3.38 log_10_ CFU/mL and 3.11 to 3.01 log_10_ CFU/mL, respectively.

Among the dual-drug combinations, the CFU value of the BDQ-DLM treatment group was lower than that of the BDQ-Pa824 treatment group. Among the three-drug combinations, the combinations of LZD and BDQ with Pa824 or DLM showed similar CFU values from 1 day to 7 days of treatment. Specifically, in the combinations of CFZ and BDQ with Pa824 or DLM, the BDQ-DLM-CFZ treatment group exhibited a lower CFU value than the BDQ-Pa824-CFZ treatment group. In summary, the above results showed that single-drug treatments (LZD or CFZ) and dual-drug combination treatments (BDQ and DLM, BDQ and Pa824) demonstrated moderate inhibitory effects.

The results indicated that neither single-drug treatments (LZD or CFZ) nor dual-drug combinations (BDQ and DLM, BDQ and Pa824) demonstrated significant antibacterial effects. Furthermore, a progressive reduction in antibacterial efficacy was noted from day 1 to day 7 for both single-drug (CFZ) and dual-drug combination treatments (BDQ and Pa824). Only the three-drug combinations sustained significant synergy effects, notably BDQ-Pa824-LZD and BDQ-DLM-LZD. As is well known, BDQ-Pa824-LZD is the combination recommended by the WHO. In this study, no statistically significant difference in efficacy was observed between the BDQ-DLM-LZD and BPaL regimens, which suggests that BDQ-DLM-LZD could be considered a potential therapeutic alternative for MDR/RR-TB.

It should be noted that although we chose 7 days as the observation period to capture the early bactericidal activity of the drug combination, in order to more reliably evaluate the persistence of the synergistic effect and prevent the regeneration of drug-resistant bacteria, we will carry out longer bactericidal experiments in the future to verify.

## DISCUSSION

This study aims to evaluate the synergistic effects of novel antituberculosis drug combinations against drug-resistant *M. tuberculosis in vitro*. Our results demonstrated that both dual-drug (BDQ-DLM and BDQ-Pa824) and triple-drug combinations (BDQ-DLM-LZD, BDQ-DLM-CFZ, BDQ-Pa824-LZD, and BDQ-Pa824-CFZ) exhibited significant antibacterial activity, with triple-drug regimens showing sustained synergistic effects over time.

In dual-drug combinations, BDQ-Pa824 showed a higher synergistic rate (43.3%) compared to BDQ-DLM (36.7%), although both combinations significantly reduced the MIC50 and MIC90 values of the constituent drugs. This suggests that while both nitroimidazoles synergize effectively with BDQ, Pa824 may have a marginally superior synergistic profile *in vitro*. However, neither dual-drug combination exhibited antagonism, supporting their compatibility in clinical regimens.

Notably, in triple-drug combinations, the addition of LZD or CFZ to BDQ-DLM or BDQ-Pa824 further enhanced antibacterial efficacy. The combinations BDQ-DLM-LZD and BDQ-Pa824-LZD showed comparable synergistic effects, with no statistically significant differences in FICI values or CFU reduction. This finding is clinically relevant, as it suggests that BDQ-DLM-LZD could serve as a potential alternative to the WHO-recommended BPaL (BDQ-Pa824-LZD) regimen, particularly in settings where Pa824 is unavailable or contraindicated.

The CFU assay further corroborated these findings. While single-drug and dual-drug treatments showed limited or transient antibacterial effects, triple-drug combinations—especially those containing LZD—demonstrated sustained bacterial suppression over 7 days. The BDQ-DLM-LZD regimen, in particular, maintained low CFU counts throughout the observation period, indicating robust and durable bactericidal activity.

Comparing the findings of this study with previously published comprehensive research holds significant value. An earlier study that integrated computer simulations, *in vitro*, and *in vivo* models demonstrated the potent antibacterial potential of the BDQ-centered triple therapy regimen (21), consistent with our results. Our study builds upon prior *in vitro* and computational models that highlighted the promise of BDQ-centered triple therapy. However, unlike previous studies that often relied on laboratory-adapted strains, our use of clinically isolated MDR/RR-TB strains enhances the translational relevance of our findings. The consistent synergy observed across diverse genetic backgrounds supports the potential broad applicability of these regimens in complex drug-resistant TB cases (13, 22).

Despite these promising results, several limitations must be acknowledged. The *in vitro* nature of this study and the relatively small sample size from a single center limit the generalizability of our conclusions. Moreover, the 7-day observation period, while sufficient to demonstrate early bactericidal activity, may not fully capture the long-term efficacy or resistance development potential of these regimens. Future studies should incorporate longer-term time-kill assays, molecular resistance profiling, and *in vivo* models to validate these findings and assess pharmacokinetic-pharmacodynamic correlations.

In light of the growing concern over cross-resistance—particularly involving *Rv0678* mutations affecting both BDQ and CFZ, and *fbi* operon mutations conferring resistance to nitroimidazoles—the strategic selection of drug combinations must be guided by local resistance patterns and individual patient drug susceptibility profiles. For BDQ, the primary resistance mechanism involves mutations in the Rv0678 gene, which encodes a repressor of the MmpS5-MmpL5 efflux pump (23). Mutations in this gene lead to the pump’s overexpression, reducing intracellular drug accumulation and conferring cross-resistance to CFZ (12).

For the nitroimidazoles DLM and Pa824, resistance primarily arises from mutations in genes involved in the F420-dependent bio-activation pathway, such as *ddn*, *fgd1*, *fbiA*, *fbiB*, *fbiC*, and *fbiD* (24–26). Regarding LZD, a critical oxazolidinone for MDR-TB treatment, resistance is mainly associated with mutations in the 23S rRNA gene (*rrl*) and ribosomal protein L3 (*rplC*), such as T460C (Cys154Arg) and *rrl* G2814T (27). The emergence of LZD resistance under treatment pressure, with some mutants exhibiting very high MICs (e.g., >16 mg/L), highlights the necessity for therapeutic drug monitoring, especially considering its dose-limiting hematological toxicities are now linked to its metabolite PNU142586 inhibiting DNA topoisomerase TOP2A/TOP2B (28).

Our study provides compelling preliminary evidence that BDQ-DLM-LZD represents a viable alternative to BPaL, particularly in settings where Pa824 access is limited. As resistance patterns continue to evolve, the strategic preservation of these core antituberculosis agents will depend on vigilant resistance monitoring, robust drug susceptibility testing capabilities, and thoughtful regimen selection guided by local resistance epidemiology.

### Conclusion

In conclusion, this study provides *in vitro* evidence supporting the therapeutic potential of the BDQ-DLM-LZD regimen as a viable alternative to BPaL for the treatment of MDR/RR-TB. Further validation in animal models and clinical trials is essential to confirm its efficacy, safety, and feasibility in real-world settings.

## SUPPLEMENTAL MATERIAL

Tables S1 to S5 spectrum.02128-25-s0001.docx Comparison of MICs, FICI, and CFU for dual-drug or triple-drug combinations.

ASM does not own the copyrights to Supplemental Material that may be linked to, or accessed through, an article. The authors have granted ASM a non-exclusive, world-wide license to publish the Supplemental Material files. Please contact the corresponding author directly for reuse.

## References

[1] Dartois VA, Rubin EJ. 2022. Anti-tuberculosis treatment strategies and drug development: challenges and priorities. Nat Rev Microbiol 20:685–701. doi:10.1038/s41579-022-00731-y 35478222 PMC9045034

[2] World Health Organization. 2025. Global tuberculosis report 2025. Geneva World Health Organization. https://www.who.int/publications/i/item/9789240116924.

[3] Maier C, Chesov D, Schaub D, Kalsdorf B, Andres S, Friesen I, Reimann M, Lange C. 2023. Long-term treatment outcomes in patients with multidrug-resistant tuberculosis. Clin Microbiol Infect 29:751–757. doi:10.1016/j.cmi.2023.02.013 36842637

[4] Chen J, Ekiert DC. 2023. A tale of two inhibitors: diarylquinolines and squaramides. EMBO J 42:e114912. doi:10.15252/embj.2023114912 37435707 PMC10390866

[5] Rashitov M, Franke MF, Trevisi L. 2024. Safety and effectivenand effectiveness of 3 novel all-oral shortened regimens for rifampicin- or multidrug-resistant tuberculosis inNovel all-oral shortened regimens for rifampicin- or multidrug-resistant tuberculosis in Kazakhstan. Clin Infect Dis 79:1046–1053. doi:10.1093/cid/ciae305 38833593 PMC11478590

[6] Guo Y, Yang J, Wang W, Wu X, Wan B, Wang H, Sha W, Yu F. 2023. Bedaquiline, delamanid, linezolid, clofazimine, and capreomycin MIC distributions for drug resistance *Mycobacterium tuberculosis* in Shanghai, China. Infect Drug Resist 16:7587–7595. doi:10.2147/IDR.S440711 38107433 PMC10723587

[7] Mirzayev F, Viney K, Linh NN, Gonzalez-Angulo L, Gegia M, Jaramillo E, Zignol M, Kasaeva T. 2021. World Health Organization recommendations on the treatment of drug-resistant tuberculosis, 2020 update. Eur Respir J 57:03300–2020. doi:10.1183/13993003.03300-2020 PMC8176349 33243847

[8] Ahmed I, Jabeen K, Inayat R, Hasan R. 2013. Susceptibility testing of extensively drug-resistant and pre-extensively drug-resistant *Mycobacterium tuberculosis* against levofloxacin, linezolid, and amoxicillin-clavulanate. Antimicrob Agents Chemother 57:2522–2525. doi:10.1128/AAC.02020-12 23507286 PMC3716178

[9] Korotych O, Achar J, Gurbanova E, Hovhannesyan A, Lomtadze N, Ciobanu A, Skrahina A, Dravniece G, Kuksa L, Rich M, et al.. 2024. Effectiveness and safety of modified fully oral 9-month treatment regimens for rifampicin-resistant tuberculosis: a prospective cohort study. Lancet Infect Dis 24:1151–1161. doi:10.1016/S1473-3099(24)00228-7 38880112 PMC11424498

[10] Guglielmetti L, Khan U, Velásquez GE, Gouillou M, Abubakirov A, Baudin E, Berikova E, Berry C, Bonnet M, Cellamare M, et al.. 2025. Oral regimens for rifampin-resistant, fluoroquinolone-susceptible tuberculosis. N Engl J Med 392:468–482. doi:10.1056/NEJMoa2400327 39879593 PMC7617355

[11] Lange C, Vasiliu A, Mandalakas AM. 2023. Emerging bedaquiline-resistant tuberculosis. Lancet Microbe 4:e964–e965. doi:10.1016/S2666-5247(23)00321-X 37931639 PMC11460056

[12] Sonnenkalb L, Carter JJ, Spitaleri A, Iqbal Z, Hunt M, Malone KM, Utpatel C, Cirillo DM, Rodrigues C, Nilgiriwala KS, Fowler PW, Merker M, Niemann S, Comprehensive Resistance Prediction for Tuberculosis: an International Consortium. 2023. Bedaquiline and clofazimine resistance in *Mycobacterium tuberculosis*: an *in-vitro* and *in-silico* data analysis. Lancet Microbe 4:e358–e368. doi:10.1016/S2666-5247(23)00002-2 37003285 PMC10156607

[13] Martin A, Bland MJ, Rodriguez-Villalobos H, Gala JL, Gabant P. 2023. Promising antimicrobial activity and synergy of bacteriocins agaiing antimicrobial activity and synergy of bacteriocins against *Mycobacterium tuberculosis*. Microb Drug Resist 29:165–174. doi:10.1089/mdr.2021.0429 35852864

[14] Gao T, Yao C, Shang Y, Su R, Zhang X, Ren W, Li S, Shu W, Pang Y, Li Q. 2023. Antimicrobial effect of oxazolidinones and its synergistic effect with bedaquiline against *Mycobacterium abscessus* complex. Infect Drug Resist 16:279–287. doi:10.2147/IDR.S395750 36683910 PMC9850832

[15] Nguyen TQ, Hanh BTB, Jeon S, Heo BE, Park Y, Choudhary A, Moon C, Jang J. 2022. Synergistic effect of Q203 combined with PBTZ169 against *Mycobacterium tuberculosis*. Antimicrob Agents Chemother 66:e0044822. doi:10.1128/aac.00448-22 36321819 PMC9765072

[16] Mansjö M, Espinosa-Gongora C, Samanci I, Groenheit R, Werngren J. 2025. Performance of a broth microdilution assay for routine minimum inhibitory concentration determination of 14 anti-tuberculous drugs against the *Mycobacterium tuberculosis* complex based on the EUCAST reference protocol. Antimicrob Agents Chemother 69:e0094624. doi:10.1128/aac.00946-24 39692505 PMC11823602

[17] Clinical and Laboratory Standards Institute (CLSI). 2024. Performance standards for susceptibility testing of mycobacteria, nocardiae, and other aerobic actinomycetes. In CLSI standard M24, 7th ed. Clinical and Laboratory Standards Institute. 31339680

[18] Köser CU, Miotto P, Ismail N, Anthony RM, Utpatel C, Merker M, Niemann S, Tahseen S, Rigouts L, Rodrigues C, Omar SV, Farhat MR, Antonenka U, Hoffmann H, Cirillo DM, Schön T. 2024. A composite reference standard is needed for bedaquiline antimicrobial susceptibility testing for *Mycobacterium tuberculosis* complex. Eur Respir J 64:2400391. doi:10.1183/13993003.00391-2024 38991722 PMC11237371

[19] Clinical and Laboratory Standards Institute (CLSI). 2018. Performance standards for susceptibility testing of mycobacteria, nocardia spp., and other aerobic actinomycetes. In CLSI Supplement M62, 1st ed 31339680

[20] EUCAST. 2023. Breakpoint tables for interpretation of MICs and zone diameters. Version 13.0. 2023.

[21] Singh R, Ramachandran V, Shandil R, Sharma S, Khandelwal S, Karmarkar M, Kumar N, Solapure S, Saralaya R, Nanduri R, Panduga V, Reddy J, Prabhakar KR, Rajagopalan S, Rao N, Narayanan S, Anandkumar A, Balasubramanian V, Datta S. 2015. *In silico*-based high-throughput screen for discovery of novel combinations for tuberculosis treatment. Antimicrob Agents Chemother 59:5664–5674. doi:10.1128/AAC.05148-14 26149995 PMC4538536

[22] Ying R, Huang X, Gao Y, Wang J, Liu Y, Sha W, Yang H. 2021. *In vitro* synergism of six antituberculosis agents against drug-resistant *Mycobacterium tuberculosis* isolated from retreatment tuberculosis patients. Infect Drug Resist 14:3729–3736. doi:10.2147/IDR.S322563 34548797 PMC8449861

[23] Rupasinghe P, Ismail N, Mulders W, Warren RM, Joseph L, Ngcamu D, Gwala T, Omar SV, Vereecken J, de Jong BC, Rigouts L, Borroni E, Cirillo DM, Schön T, Miotto P, Köser CU. 2025. *In vitro* exposure to clofazimine can select for delamanid and pretomanid resistance in *Mycobacterium tuberculosis*. Antimicrob Agents Chemother 69:e0111325. doi:10.1128/aac.01113-25 40980917 PMC12587575

[24] Liu Y, Shi J, Li L, Wu T, Chu P, Pang Y, Guo Y, Gao M, Lu J. 2022. Spontaneous mutational patterns and novel mutations for delamanid resistance in *Mycobacterium tuberculosis*. Antimicrob Agents Chemother 66:e0053122. doi:10.1128/aac.00531-22 36448833 PMC9765178

[25] Nguyen TVA, Nguyen QH, Nguyen TNT, Anthony RM, Vu DH, Alffenaar J-WC. 2023. Pretomanid resistance: An update on emergence, mechanisms and relevance for clinical practice. Int J Antimicrob Agents 62:106953. doi:10.1016/j.ijantimicag.2023.106953 37595848

[26] Reichmuth ML, Hömke R, Zürcher K, Sander P, Avihingsanon A, Collantes J, Loiseau C, Borrell S, Reinhard M, Wilkinson RJ, Yotebieng M, Fenner L, Böttger EC, Gagneux S, Egger M, Keller PM. 2020. Natural polymorphisms in *Mycobacterium tuberculosis* conferring resistance to delamanid in drug-naive patients. Antimicrob Agents Chemother 64:00513–00520. doi:10.1128/AAC.00513-20 PMC7577131 32868333

[27] Kim Y, Lee D-G, Bae J, Jung J, Jeon H, Chung M, Park J-A, Ahn K, Mun J-W, Jang Y, Lee H, Jang J, Ryoo S. 2026. Characteristic exploration of *Mycobacterium tuberculosis* isolated from patients with linezolid-treated multidrug-resistant tuberculosis. Eur J Clin Microbiol Infect Dis 45:429–440. doi:10.1007/s10096-025-05319-x 41160260

[28] Thu VTA, Nhu NQ, Anh NTV, Lim S-A, Seong H-J, Md Rasheduzzaman J, Kim U, Cho H-S, Soedarsono S, Shin J-G, Cho Y-S. 2025. Deciphering linezolid-induced hematologic toxicity: Targeting TOP2A and TOP2B via its primary metabolite PNU142586. Sci Adv 11:eadt5833. doi:10.1126/sciadv.adt5833 40435237 PMC12118551

