# Supplementary material for: Synergism in drug combination of new tuberculosis antibiotics for drug-resistant Mycobacterium tuberculosis in vitro
Source: Microbiol Spectr. 2026 Jul 10;14(8):e02128-25. doi: 10.1128/spectrum.02128-25 (PMC13436375; doi:10.1128/spectrum.02128-25)
Supplement: Supplementary file 1 [file spectrum.02128-25-s0001.docx]

**SUPPLEMENTAL MATERIAL**

[Supplementary Table 1](https://static-content.springer.com/esm/art:10.1038/s41586-022-05114-0/MediaObjects/41586_2022_5114_MOESM1_ESM.docx)

Comparison of MICs for Each Drug Before and After Combinations from Dual-Drug Susceptibility Testing

[Supplementary Table 2](https://static-content.springer.com/esm/art:10.1038/s41586-022-05114-0/MediaObjects/41586_2022_5114_MOESM1_ESM.docx)

Distribution and Comparison of FICI of 30 Strains from Two Dual-Drug Combinations

[Supplementary Table 3](https://static-content.springer.com/esm/art:10.1038/s41586-022-05114-0/MediaObjects/41586_2022_5114_MOESM1_ESM.docx)

Comparison of MICs for Each Drug Before and After Combinations from Triple-Drug Susceptibility Testing

[Supplementary Table 4](https://static-content.springer.com/esm/art:10.1038/s41586-022-05114-0/MediaObjects/41586_2022_5114_MOESM1_ESM.docx)

Distribution and Comparison of FICI of 30 Strains from Four Triple-Drug Combinations

[Supplementary Table 5](https://static-content.springer.com/esm/art:10.1038/s41586-022-05114-0/MediaObjects/41586_2022_5114_MOESM1_ESM.docx)

The CFU Values of Drug Combinations at Different Treatment Times

**Table S1** Comparison of MICs for Each Drug Before and After Combinations from Dual-Drug Susceptibility Testing

| **Drug Combination** | **MIC (Mg/Liter)** | | | ***P* value** |
| --- | --- | --- | --- | --- |
|  | **MIC_50_** | **MIC_90_** | **Range** |  |
| **BDQ-DLM** |  |  |  |  |
| bdq | 0.06 | 0.5 | 0.015 to 1 |  |
| BDQ | 0.125 | 1 | 0.03 to 1 | **<0.001** |
| dlm | 0.0075 | 0.06 | 0.0015 to 1 |  |
| DLM | 0.03 | 4 | 0.0075 to 4 | **<0.001** |
| **BDQ-Pa824** |  |  |  |  |
| bdq | 0.03 | 0.5 | 0.015 to 1 |  |
| BDQ | 0.125 | 1 | 0.03 to 1 | **<0.001** |
| pa824 | 0.0015 | 0.03 | 0.0015 to 0.5 |  |
| Pa824 | 0.03 | 4 | 0.003 to 4 | **<0.001** |

**Notes**: BDQ, MIC of BDQ tested alone; bdq, MIC of BDQ tested with the combination of DLM or Pa824; DLM, MIC of DLM tested alone; dlm, MIC of DLM tested with the combination of BDQ; Pa824, MIC of Pa824 tested alone; pa824, MIC of Pa824 tested with the combination of BDQ; data in bold indicate that the *P* values were statistically significant.

**Table S2** Distribution and Comparison of FICI of 30 Strains from Two Dual-Drug Combinations

| **FICI Value** | **BDQ-DLM^a^** | **BDQ-Pa824^b^** |
| --- | --- | --- |
| ≤0.5  0.5< and ≤4  >4 | 11(36.7%)^c^ | 13(43.3%) |
|  | 19(63.3%) | 17(56.7%) |
|  | 0 | 0 |

**Notes**: *P* value for a vs b comparison was 0.0393; c, the data in and outside the

brackets represented the percentages and frequencies.

**Table S3** Comparison of MICs for Each Drug Before and After Combinations from Triple-Drug Susceptibility Testing

| **Drug Combination** | **MIC (Mg/Liter)** | | | ***P* value** |
| --- | --- | --- | --- | --- |
|  | **MIC_50_** | **MIC_90_** | **Range** |  |
| **BDQ-DLM-LZD** |  |  |  |  |
| bdq | 0.03 | 0.03 | 0.03 |  |
| dlm | 0.0015 | 0.0015 | 0.0015 |  |
| lzd | 0.25 | 2 | 0.25-2 |  |
| LZD | 0.5 | 8 | 0.5-16 | **<0.001** |
| **BDQ-DLM-CFZ** |  |  |  |  |
| bdq | 0.03 | 0.03 | 0.03 |  |
| dlm | 0.0015 | 0.0015 | 0.0015 |  |
| cfz | 0.125 | 0.5 | 0.125-0.5 |  |
| CFZ | 0.06 | 4 | 0.06-8 | **<0.001** |
| **BDQ-Pa824-LZD** |  |  |  |  |
| bdq | 0.03 | 0.03 | 0.03 |  |
| pa824 | 0.0015 | 0.0015 | 0.0015 |  |
| lzd | 0.25 | 2 | 0.25-2 |  |
| LZD | 0.5 | 8 | 0.5-16 | **<0.001** |
| **BDQ-Pa824-CFZ** |  |  |  |  |
| bdq | 0.03 | 0.03 | 0.03 |  |
| pa824 | 0.0015 | 0.0015 | 0.0015 |  |
| cfz | 0.125 | 0.5 | 0.125-0.5 |  |
| CFZ | 0.06 | 4 | 0.06-8 | **<0.001** |

**Notes**: bdq, MIC of BDQ tested with the combination of DLM or Pa824 with CFZ or LZD; dlm, MIC of DLM tested with the combination of BDQ and LZD or BDQ and CFZ; pa824, MIC of Pa824 tested with the combination of BDQ and LZD or BDQ and CFZ; LZD, MIC of LZD tested alone; lzd, MIC of LZD tested with the combination of BDQ and DLM or BDQ and Pa824; CFZ, MIC of CFZ tested alone; cfz, MIC of CFZ tested with the combination of BDQ and DLM or BDQ and Pa824; data in bold indicate that the *P* values were statistically significant.

**Table S4** Distribution and Comparison of FICI of 30 Strains from Four Triple-Drug Combinations

| **FICI Value** | **BDQ-DLM-LZD^a^** | **BDQ-DLM-CFZ^b^** | **BDQ-Pa824-LZD^c^** | **BDQ-Pa824-CFZ^d^** |
| --- | --- | --- | --- | --- |
| ≤0.75  0.75< and ≤4  >4 | 19(63.3%)^e^ | 19(63.3%) | 19(63.3%) | 19(63.3%) |
|  | 11(36.7%) | 11(36.7%) | 11(36.7%) | 11(36.7%) |
|  | 0 | 0 | 0 | 0 |

**Notes**: *P* value for a vs b comparison was 0.9476; *P* value for a vs c comparison was＞0.9999; *P* value for a vs d comparison was 0.9292; *P* value for b vs c comparison was 0.9531; *P* value for b vs d comparison was＞0.9999; *P* value for c vs d comparison was 0.9358; e, the data in and outside the brackets represented the percentages and frequencies.

**Table S5** The CFU Values of Drug Combinations at Different Treatment Times

| **Drug Combination** | **Treatment Time (Days) / Log_10_ CFU/mL** | | |
| --- | --- | --- | --- |
|  | **0 day** | **1 day** | **7 days** |
| **NC** | 4.00 | 3.65 | 5.25 |
| **LZD** | 4.00 | 3.56 | 3.48 |
| **CFZ** | 4.00 | 3.34 | 3.88 |
| **BDQ-DLM** | 4.00 | 3.03 | 3.04 |
| **BDQ-Pa824** | 4.00 | 3.21 | 3.58 |
| **LZD-BDQ-Pa824** | 4.00 | 3.18 | 2.63 |
| **LZD-BDQ-DLM** | 4.00 | 3.29 | 2.66 |
| **CFZ-BDQ-Pa824** | 4.00 | 3.41 | 3.38 |
| **CFZ-BDQ-DLM** | 4.00 | 3.11 | 3.01 |
